# Erysipelas, a large retrospective study of aetiology and clinical presentation

**DOI:** 10.1186/s12879-015-1134-2

**Published:** 2015-09-30

**Authors:** Anna Bläckberg, Kristina Trell, Magnus Rasmussen

**Affiliations:** Division of Infection Medicine, Department of Clinical Sciences Lund, Lund University, Lund, Sweden

**Keywords:** Erysipelas, Bacteraemia, Aetiology, Beta-haemolytic streptococci

## Abstract

**Background:**

Erysipelas is a common and severe infection where the aetiology and optimal management is not well-studied. Here, we investigate the clinical features, bacteriological aetiology, and treatment of erysipelas.

**Methods:**

Episodes of erysipelas in a seven-years period in our institution were studied retrospectively using a pre-specified protocol and is presented with descriptive and comparative statistics.

**Results:**

1142 episodes of erysipelas were identified in 981 patients. Patients had a median age of 61 years, 59 % were male, a majority had underlying diseases or predisposing conditions, and the leg was most often affected. Wound cultures were taken in 343 episodes and 56 grew group A streptococci (GAS), 53 grew group G streptococci (GGS), 11 grew group C streptococci (GCS), and 153 grew *Staphylococcus aureus.* Blood cultures were drawn in 49 % of episodes and 50 cultures were positive with GGS as the most common finding (21 cultures) followed by GAS in 13, group B streptococci in 5, *S. aureus* in 4, and GCS in 3 cultures. In 45 % of episodes, patients received antibiotics with activity against *S. aureus.*

**Conclusions:**

GGS is the most common streptococcus isolated in erysipelas and the role of *S. aureus* in erysipelas remains elusive.

## Background

Erysipelas is a common skin infection causing significant morbidity in patient which often have underlying conditions [1, 2]. The clinical picture is characterized by an inflammatory reaction of the upper dermis with a sharp demarcation of the erythema. Occasionally, a primary lesion such as a wound or skin crack is present. Associated symptoms and signs are nausea, pain, and fever [3]. Erysipelas cannot always be distinctly separated from cellulitis, which refers to a deeper soft tissue infection involving the dermis and subcutaneous fat, or from more severe conditions such as necrotizing fasciitis [4, 5]. Some clinicians use the term erysipelas only for facial cutaneous infections whereas yet others use the term erysipelas also to describe cellulitis [5]. The diagnosis of soft tissue infections relies on the clinical picture and on the diagnostic traditions, making comparisons between studies problematic.

Despite erysipelas being common, and the skin is accessible for bacteriological sampling, the aetiology of erysipelas is still not firmly established. Most authors agree that beta-haemolytic streptococci (BHS) are causative pathogens, though such bacteria are typically isolated only from a minority of patients [4]. Cultures from needle aspirates or punch biopsies of the inflamed skin identify pathogenic bacteria in a minority of cases [6–9] and cultures from primary lesions have a similar sensitivity. In three prospective studies on erysipelas, group A streptococci (GAS) were identified in 15–22 %, group G streptococci (GGS) in 3–12 %, and *Staphylococcus aureus* in pure culture in 7–18 % of cases [6, 10, 11]. In support for BHS aetiology of erysipelas, one study using immunofluorescence identified BHS in 19, of which 13 were GAS, of 27 erysipelas cases [12]. Serological studies have also been performed and support BHS aetiology in a share of the cases but results have been somewhat conflicting [6, 7, 12, 13]. Bacteraemia is rare in erysipelas and current guidelines do not recommend blood cultures to be taken in uncomplicated cases [4]. In a recent systematic review of five studies on erysipelas, 28 of 607 blood cultures (4,6 %) were positive. Of the positive cultures, 13 grew GAS, 8 other BHS, 4 *S. aureus*, and 3 gram-negatives [14].

Traditionally, BHS have been characterized by grouping and the group is not always in concordance with the species. Importantly, BHS of groups C and G streptococci (GCS and GGS) can be of different species, but lately it has become apparent that human pathogenic GGS are nearly always *Streptococcus dysgalactiae subspecies equisimilis* (SDSE) [15]. GCS are also commonly SDSE but *Streptococcus equi subspecies zooepidemicus* of group C also cause human infections [16]. Human pathogenic GAS (which is nearly always *Streptococcus pyogenes*) and SDSE share many important virulence strategies and are both uniformly sensitive to penicillin [17].

The lack of uniform diagnostic criteria for erysipelas and the lack of a well-supported knowledge of its aetiology are problematic. In particular, the role of *S. aureus* in erysipelas is uncertain and many patients with erysipelas are probably therefore treated with antibiotics with an unnecessary broad spectrum. In this retrospective analysis, we present the clinical and microbiological findings from patients with erysipelas treated at our clinic between 2007 and 2013.

## Methods

Cases of patients ≥18 years of age registered with a ICD-10 diagnosis of erysipelas (A46.9) or cellulitis (L03) at the Department of Infectious Diseases at Skåne University Hospital, Lund, Sweden from January 1^st^ 2007 to December 31^st^ 2013 were identified. Both hospitalized and outpatients were included. The University Hospital of Lund serves approximately 200 000 inhabitants living in the surrounding area. The medical records were reviewed according to a pre-specified protocol. Microsoft Excel 2008 (Microsoft Corporation) was used for data collection and descriptive analyses and Graph Pad Prism 6.0a (GraphPad software) for statistical analyses. The Ethics Committee of Lund University, Sweden approved of the study (2011/672). The medical records regarding episodes of erysipelas between January 2007 and December 2010 had been reviewed in a previous set and an analysis of risk factors for recurrence has already been studied [2].

Epidemiological and clinical parameters recorded included age, sex, hospital stay, relapses, cases with outpatient and inpatient treatment respectively, localisation of erysipelas, antibiotic treatment and duration, the criteria severe inflammatory response syndrome (SIRS) [18], CRP, creatinine, and platelet levels on admission. Underlying disease was classified as cardiovascular disease, autoimmune/systemic inflammatory disease, cancer, diabetes mellitus, chronic obstructive pulmonary disease (COPD), or kidney disease. Predisposing factors were defined as having wounds, previous radiation therapy, local operation (any kind), peripheral venous insufficiency, polyneuropathy, lymphedema, or skin disease. Microbiological results from swabs and from blood cultures were collected from the accredited Laboratory for Medical Microbiology in Lund. Isolates of BHS had previously been grouped by the routine laboratory. We subjected BHS isolates from blood to matrix-assisted laser desorption ionization time-of-flight mass spectrometry (MALDI-TOF) with the direct transfer method and analysis with Ultraflextreme MALDI-TOF MS (Bruker Daltonics, Bremen, Germany), using the Biotyper version 3.0 software without modifications.

### Statistics

Fisher’s exact test and Mann Whitney test were utilized to detect statistically differences between different groups depending on the lighted variables. Significance was defined as a *P*-value less than 0.05.

## Results

### Patients

1142 episodes of erysipelas in 981 patients were recorded and studied. During the same time period 188 episodes of cellulitis were recorded. The diagnosis of “cellulitis” is in our Department used to describe a deeper type of infection and these cases were not further studied. The patients were predominately male (59 %) and the median age was 61 (range 18–99 years). 44 % of patient had an underlying disease and 78 % had one or several predisposing factors. Only 13 % of the patients had neither predisposing factors nor an underlying disease (Table 1). In 567 episodes, patients were treated as outpatients and in 575 episodes the patient was admitted. The median length of stay for admitted patients was 5 days (range 1–34 days). 745 episodes were first-time infections and 397 represented relapses. The most common location of erysipelas was in the leg (66 %) followed by arm, and face (Table 2). 39 % of all cases fulfilled the criteria for SIRS, and median CRP and WBC count upon evaluation was 76 and 10.6 respectively (Table 2).

**Table 1:** Characteristic of 981 patients with erysipelas

Clinical characteristic	No. patients (%)
Age, median (range)	61 (18–99)
Male sex	580 (59 %)
Underlying disease	433 (44 %)
Predisposing factor	766 (78 %)
Both underlying disease and predisposing factor	346 (35 %)
Neither underlying disease nor predisposing factor	128 (13 %)

**Table 2:** Characteristic of 1142 episodes of erysipelas

Site of infection	
Leg	771 (68 %)
Arm/hand	136 (12 %)
Face	107 (9 %)
Other	128 (11 %)
Previous erysipelas	397 (35 %)
Episodes with SIRS (≥2points)	444 (39 %)
CRP (mg/L), mean (range)	76 (<0.6–520)
Leukocyte count (X10^/L), mean (range)	10,6 (2–326)
Temperature C°, mean (range)	37, 3 (35.1–41)
Days of hospitalization, median (range)	5 (1–34)
Days of antibiotic treatment, median (range)	11 (1–29)
Outpatient treatment	567 (50 %)
Initial treatment with iv antibiotics	713 (62 %)
Definite treatment	
Penicillin	569 (50 %)
Antibiotic effective against S. aureus	461 (40 %)
Allergy to penicillin	112 (10 %)

### Wound cultures

**Fig. 1:**
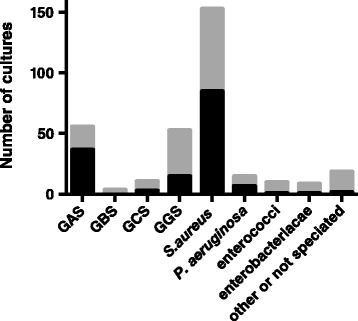
Number of skin swabs and wound cultures positive for different possible pathogens is given. The black part of the bar represents a monoculture of the respective pathogen whereas the grey part of the bar represents culture where more than one bacterial species was identified

A wound culture was taken in 343 of the 1142 episodes and of the cultures 248 (72 %) grew bacteria. Patients from which such cultures were taken were significantly older (63 vs 60 years), were more often admitted, and a had more often predisposing conditions or underlying diseases as compared to cases where no skin culture was taken. Of the positive cultures 56 (23 %) grew GAS of which 37 cultures were monocultures, 53 (21 %) grew GGS of which 15 cultures were pure, 11 grew a BHS of group C (GCS) of which 3 were in pure cultures, and 153 (62 %) grew *S. aureus* of which 85 cultures were pure (Fig. 1). None of the *S. aureus* isolates were resistant to methicillin. Sixteen cultures were polymicrobial and bacteria were not species determined and another 15 cultures (6 %) grew *Pseudomonas aeruginosa*. Enterococci were encountered in 10 cultures (4 %) and enterobacteriacae in 9 cultures (4 %) (Fig. 1).

Notably, a significantly higher proportion of GGS and GCS than GAS isolates were found in co-cultures with *S. aureus* (72 vs 30 %, *p* < 0.0001 with Fischer’s exact test for a difference between GGS and GAS). Three patients with GAS and three patients with GGS in co-cultures with *S. aureus* had bacteraemia with the respective BHS.

### Blood cultures

Blood cultures were collected in 555 episodes (49 %). Of obtained cultures, 492 (89 %) were negative, 13 (2 %) were considered to be contaminated (finding of coagulase negative staphylococci in one or two flasks), and 50 (9 %) were positive. Patients subjected to blood culturing were significantly more likely to fulfil criteria for SIRS (55 vs 23 %), to be admitted (79 vs 23 %), to have underlying diseases (52 vs 41 %) particularly diabetes mellitus (18 vs 11 %), and to be of male sex (63 vs 57 %) as compared to the cases where no blood culture was taken. In addition, patients where blood cultures were taken had a significantly higher mean CRP (91 vs 57 mg/L) and were more often subjected to wound cultures (64 vs 25 %).

**Fig. 2:**
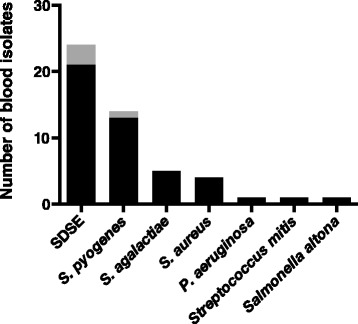
Number of blood cultures positive for different bacterial species. For SDSE, the black part of the bar represents GGS and the grey part represents GCS. For *S. pyogenes*, the black part of the bar represents monocultures whereas the grey part represents a culture which also grew *P. aeruginosa* and coagulase negative staphylococci

The results of the blood cultures are shown in Fig. 2. The most commonly isolated bacterium in blood was GGS (21 cultures). The GGS isolates as well as the three GCS isolates were determined to be *Streptococcus dysgalactiae* using MALDI-TOF MS. GAS (confirmed to be *Streptococcus pyogenes* with MALDI-TOF MS) was isolated in 13 cases, GBS (confirmed to be *Streptococcus agalactiae* with MALDI-TOF MS) in five cases, and *S. aureus* in four cases. Patients with *S. pyogenes* bacteraemia were younger than patients with SDSE bacteraemia (63 vs 73 years, *p* = 0.02 for difference using Wilcoxon’s rank sum test), but otherwise clinical parameters were similar. Patients with *S. aureus* bacteraemia had septic arthritis, cellulitis, a deep soft tissue infection, and infective endocarditis respectively. In six episodes with findings of GAS in blood, a cutaneous swab also grew GAS and in five episodes with findings of GGS in blood the cutaneous swab also grew GGS. Of the four patients with *S. aureus* in blood cultures, three had findings of *S. aureus* from wound cultures.

Taken together, the results from the microbiological analyses imply GAS as the causative organism in 61 cases, GGS or GCS in 82 cases, and GBS in 9 cases. When comparing episodes where GAS was implied with those where GCS/GGS were implied, the only statistically significant differences were that GCS/GGS episodes more commonly affected male patients (74 vs 57 %,) and were more likely to have recurrence (35 vs 18 %).

### Treatment and outcome

In 643 episodes (56 %), empirical treatment was initiated with penicillin of which 435 received intravenous penicillin G. In 131 episodes the initial treatment was cloxacillin or flucloxacillin, in 195 episodes a beta-lactam antibiotic with broad spectrum, in 150 episodes clindamycin, and other antibiotics were given initially in 22 episodes. For definite treatment, defined as the antibiotic prescribed when the patient left the hospital, penicillin was used in 567 (55 %) cases and antibiotics effective against methicillin susceptible *S. aureus* (MSSA) was used in 463 (45 %) cases. Patients allergic to penicillin were excluded from this analysis. Median duration of treatment was 11 days (range 1–34).

Of cases with *S. aureus* in a microbiological specimen, 64 % received treatment with an antibiotic effective against MSSA compared to 36 % in the group where no microbiological evidence for *S. aureus* was present (*p* = 0.0001). Patients receiving an antibiotic effective against MSSA as definite treatment were more likely to have an underlying condition (82 vs 74 %, *p* = 0.001). Interestingly, patients with a previous episode of erysipelas was more likely to receive penicillin (62 vs 52 %, *p* = 0.003) whereas those receiving penicillin or MSSA effective treatment had similar risk of recurrence. Outcome was generally favourable and only four fatalities were recorded. Twenty-five patients received secondary prophylaxis with penicillin.

## Discussion

Microbiological methods to determine the aetiology of erysipelas have low sensitivity and treatment is in most cases empirical. Previous studies have mostly been prospective and relatively small. The present retrospective study is by far the largest presented and it confirms some of the previously reported findings whereas it contrasts to earlier notions on the relative importance of GAS and GCS/GGS. This study has several shortcomings. The retrospective nature does not allow us to specify how patients should be cultured or managed. We chose to include patients where the attending physician had made a diagnosis of erysipelas. Likely, different physicians have different criteria and the tradition of our clinic probably also affect the likelihood of the diagnosis. The study was carried out in a single institution to which a selected patient population might seek care, though our hospital is the only one serving the population. Probably, patients coming to our institution are more likely to be severely ill and have underlying conditions than patients coming to a primary care unit. Generalizations made from the present results should be done with care.

As in previous studies, we find that patients with erysipelas are likely to be male, around 60 years-old, and have underlying diseases and predisposing conditions. Moreover, we find that blood culture positivity is rare in erysipelas though our figure (50 positives out of 555 cultures) is significantly higher than those reported previously [14] (28 positives of 607 cultures, *p* = 0.003 for a difference using Fischers exact test). Blood cultures are not recommended in uncomplicated cases of erysipelas [4] but in a significant share of episodes (49 %) reported here a blood culture was drawn. Those subjected to blood culturing were significantly more ill than those who were not blood cultured but the sensitivity of blood cultures is still very low. Of our episodes, we found evidence for a BHS involvement in 13 % which is somewhat lower than in previous studies which have been prospective with more diagnostic efforts [6, 10, 11]. The relative contribution of different groups of BHS is different in this study compared to previous reports of erysipelas [6, 10, 12]. Importantly as opposed to previous studies, GGS was the most common BHS isolated from blood and all these isolates as well as those of GCS were shown to be *Streptococcus dysgalactiae* of which *subspecies equisimilis* is the only known human pathogen [16]. Thus, it appears that SDSE is a common and important pathogen in erysipelas. Indeed, several authors have reported an increase in severe infections with SDSE [15, 19]. An older Swedish study and several recent reports on cellulitis have also implicated GGS or SDSE as pathogens frequently causing erysipelas or cellulitis [11, 20–22]. The finding that patients with GCS/GGS aetiology were more prone to experience recurrence is very interesting and in line with a number of reports describing that recurrent bacteraemia is a particular feature of SDSE [23–26]. Indeed, an increase tendency for recurrence in GGS cellulitis as compared to GAS cellulitis was very recently demonstrated [22]. Possibly, GCS/GGS aetiology should be considered when a decision about secondary prophylaxis is made. In line with this argument, anal carriage of GGS have previously been implicated as a possible mechanism for GGS recurrence in erysipelas [27]. It is also intriguing that GGS, more often than GAS, was found together with *S. aureus* in wound cultures and this likely reflects differences in virulence and colonization strategies of the bacteria. It can be speculated that GGS is more likely to infect a person if there is a pre-existing wound colonized with *S. aureus* though such a speculation would need confirmatory studies to be supported.

As compared to earlier studies a higher proportion of wound cultures in our study grew *S. aureus* [6, 10, 11] and if this finding should be interpreted as *S. aureus* being a common causative pathogen is not clear. It was evident, however, that patients with *S. aureus* in wound cultures were more likely to receive an antibiotic effective against MSSA, suggesting that clinicians perhaps use positive *S. aureus* cultures as a basis for decision-making. *S. aureus* was found in four blood cultures but upon examination of the medical records it became evident that at least three of them had other diagnoses that should have excluded them from a diagnosis of erysipelas. Such a selected post-analysis is problematic and points to the weakness in using diagnostic codes for selection of patients. However, our results indicate that *S. aureus* bacteraemia is very uncommon in erysipelas. We cannot exclude, however, that patients with soft tissue infections and *S. aureus* bacteraemia received a diagnosis of cellulitis instead of erysipelas based on bacteriology rather than the nature of the soft tissue infection. It thus remains difficult to draw definite conclusions on the role of *S. aureus* in erysipelas. In general, the antibiotic treatment of erysipelas in our institution was directed towards a staphylococcal aetiology in large proportion of cases and this is not in line with current recommendations [4]. The median length of antibiotic treatment was 11 days, which is also longer than the suggested standard treatment time (5 days) [4].

## Conclusions

Our study indicates that GCS/GGS is an important aetiological factor in erysipelas and underlines the need for novel diagnostic procedures to better guide antibiotic therapy.
